# *De novo* Food Allergy After Pediatric Liver Transplantation: A Systematic Review

**DOI:** 10.3389/fped.2022.885942

**Published:** 2022-05-12

**Authors:** Chiara Bergamo, Emily Claire Argento, Stefania Giampetruzzi, Maristella Cutini, Francesco Ciabattoni, Giovanna Faggian, Paola Gaio, Luca Bosa, Mara Cananzi

**Affiliations:** ^1^Pediatric Residency Program, Department of Women's and Children's Health, University Hospital of Padova, Padua, Italy; ^2^Unit of Pediatric Gastroenterology, Digestive Endoscopy, Hepatology and Care of the Child With Liver Transplantation, Department of Women's and Children's Health, University Hospital of Padova, Padua, Italy

**Keywords:** liver transplant, pediatric liver transplantation, children, allergy, food allergy, *de novo* food allergy after transplant, systematic review

## Abstract

**Background:**

Liver transplant (LT) recipients, particularly children, have an increased risk of developing *de novo* food allergies (FAs) after transplantation both compared to all the other transplant groups and to the general population. Little is known about the pathogenesis underlying this phenomenon and comprehensive recommendations or clinical practice guidelines are still lacking, mainly due to the scarcity of high-quality evidence.

**Aim:**

We aimed to prepare a systematic review on *de novo* FA in pediatric LT recipients to assess epidemiology and risk factors, evaluate the correlation to specific food groups, describe clinical manifestations, investigate the rate of tolerance acquisition over time and report available therapeutic strategies.

**Methods:**

We conducted this systematic review according to the Preferred Reporting Items for Systematic Reviews and Meta-Analyses (PRISMA). MEDLINE, Scopus, Web of Science, Wiley online library, Cochrane Library, and ClinicalTrials.gov databases were systematically searched for studies published from January 1980 to September 2021. All the articles were checked independently by two reviewers in two steps. A total of 323 articles were screened, and 40 were included for data extraction.

**Results and Conclusions:**

We found that *de novo* FAs develop in the 15% of pediatric LT recipients, especially in the first 2 years after surgery, with higher risk related to younger age at transplantation (especially <2 years of age) and tacrolimus immunosuppression. Subjects are often allergic to multiple foods, and 15% of them suffer from anaphylaxis. The majority of patients do not spontaneously outgrow their symptoms during follow-up. The discontinuation of tacrolimus in favor of cyclosporine or the association of tacrolimus with mycophenolate have been associated with the resolution or the improvement of FA in small retrospective case series and could be considered in case of severe or multiple, difficult to manage FAs. Prospective multicenter studies are needed to confirm these findings, guide the risk-based stratification of pediatric LT recipients, and provide for high-evidence therapeutic strategies for children with *de novo* FA.

## Introduction

Food allergies (FAs) are defined as immune-mediated adverse reactions to food proteins caused by the lack of development or the breakdown of immunological tolerance to food (1, 2). They are broadly categorized into IgE-mediated reactions (e.g., food-induced anaphylaxis), mixed IgE and non-IgE mediated reactions (e.g., eosinophilic gastrointestinal disorders, EGID), and non-IgE-mediated (cell-mediated) reactions (e.g., food protein-induced enterocolitis syndrome, FPIES) (1).

As a consequence of the increase in the rate of organ transplantation, previously non-allergic transplant recipients have been increasingly recognized as being at higher risk for developing *de novo* FA in comparison to the general population (3–6). Moreover, children undergoing liver transplantation (LT) have resulted to be far more affected by FA when compared to all the other transplant groups (5, 7–10).

Little is known about the pathogenesis underlying the occurrence of *de novo* FA after transplantation. Two main mechanisms have been hypothesized so far. The first consists of the passive transfer of allergen-specific IgEs and immune cells from the donor to the recipient at the time of transplant. Although this mechanism has been proven in several anecdotal cases, the inherent transience of this immunological transfer does not explain the development of FAs that are absent in the donor, nor their long-term persistence after transplant (6, 11–13). The second postulated mechanism consists of a loss of immune tolerance to orally ingested food antigens induced by calcineurin inhibitors, responsible for a Th2/Th1 imbalance with predominance of Th2 over Th1 responses (4, 6, 9, 14–16).

Since the first description of a peanut allergy transferred from the donor to the recipient of a combined liver-kidney transplant in 1997 (13), there has been a growing interest regarding *de novo* FA after LT and a parallel surge in the number of published papers. However, comprehensive recommendations and clinical practice guidelines on the topic are still lacking, mainly due to the scarcity of LTs in children, acknowledged by the European Reference Network for Pediatric Transplantation, and to the absence of high-quality evidence. Thus, we conducted a systematic review on *de novo* FA in pediatric LT recipients to define epidemiology and risk factors, assess the correlation to specific food groups, describe clinical manifestations, investigate the rate of tolerance acquisition over time and report the available therapeutic strategies.

## Methods

### Search Strategy and Study Selection

This systematic review was performed in accordance with the PRISMA 2020 statement (17). The review was structured around six research questions, all relevant to the aim of the study. A bibliographic search of the literature published from January 1980 to September 2021 was performed employing the Pubmed/MEDLINE, Scopus, Web of Science, Wiley online library, Cochrane Library and ClinicalTrials.gov databases. The primary search was conducted September 15, 2021. The following search terms were used: “Food Hypersensitivity,” “Food Allergy,” “Liver Transplantation.” The detailed search strategy for PubMed is available in Supplementary Table S1.

Papers were included in the systematic review if they were in English, contained original research on human subjects aged 0 to 18 years old, and focused on *de novo* FA occurring after LT. We excluded editorials and reviews not reporting original data, studies only including patients with FA onset prior to LT, studies without relevant clinical information, and unavailable full texts.

After removal of duplicates, articles were screened by titles and abstracts. Then, selected papers were screened by full-text assessment, and their references were manually scrutinized to identify additional eligible studies. In both steps, two reviewers worked independently, then findings were merged, and discrepancies were resolved through consensus-based discussion or by involving a third investigator.

**Figure 1:**
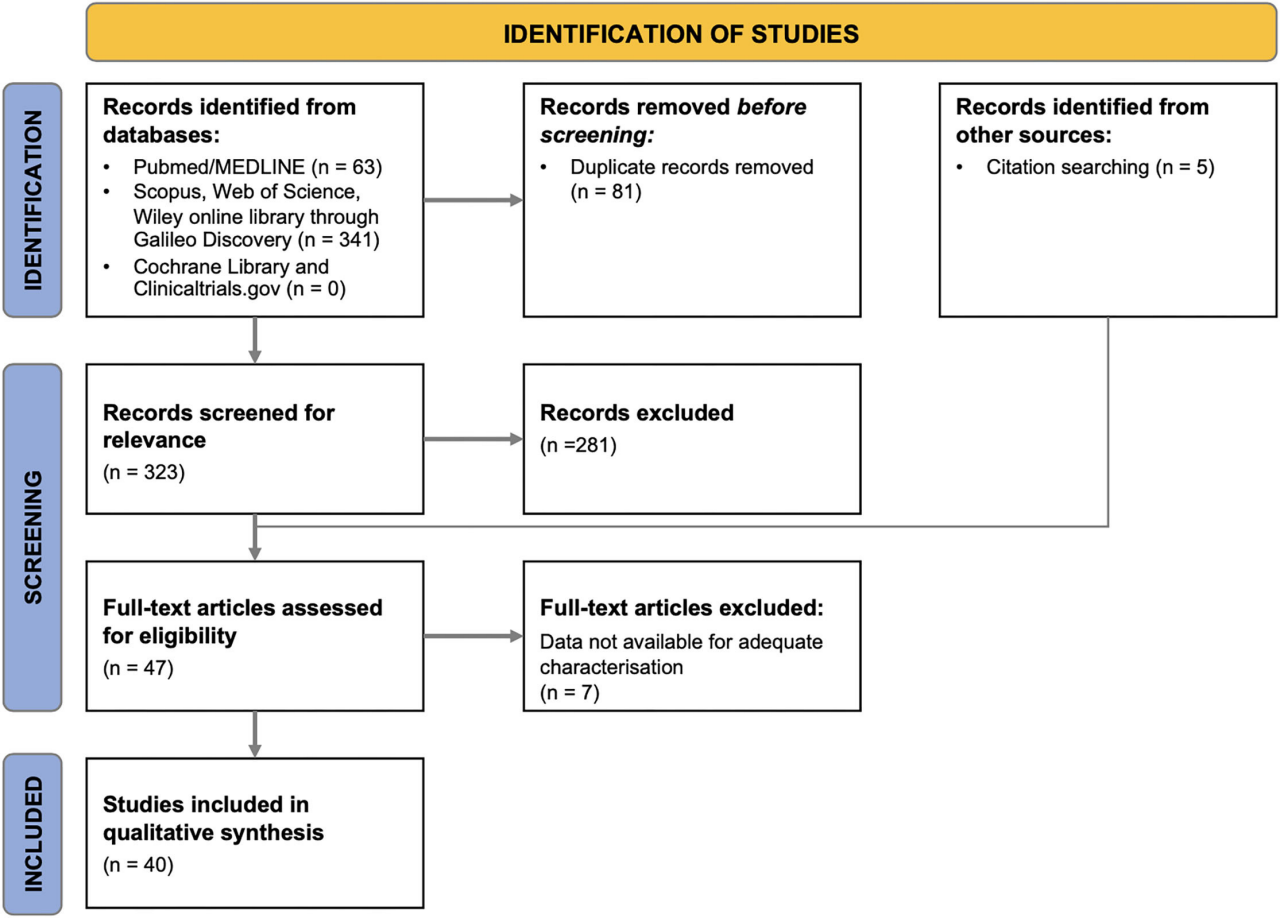
PRISMA flow diagram of the search strategy and included studies.

The flow diagram relative to the study selection process is provided in Figure 1. The studies excluded from the systematic review as well as the rationale for their exclusion are reported in Supplementary Table S2.

### Data Extraction and Quality Assessment

Following screening, two reviewers independently extracted the data from the included studies using a data extraction form (Table 1). Risk of bias for all the included studies was independently assessed by two researchers using the National Institutes of Health Quality Assessment Tools (Supplementary Table S3) (53). No studies were excluded based on quality assessment. Likewise, consecutive patient enrollment was not deemed necessary for prevalence calculations. Supplementary Table S4 specifies which studies were used to extract data to answer to each research question.

**Table 1:** Raw data obtained from the 40 studies included in the systematic review.

**N**	**Author, year**	**Study design**	**Pts with *de novo* FA/LT pts (%)**	**Female pts (%)**	**Age at LT, mos**.	**Time btw. LT and FA, mos**.	**Age at FA onset, mos**.	**Indication to LT (*n*)**	**Clinical mani festa tions**	**Culprit foods**	**Multiple FAs**	**IS at FA onset (*n*)**	**Change of IS, outcome**	**FA outgrown, n**	**FU time, mos**.
**Cohort and cross-sectional studies**															
1	Prabhakaran et al. (18)	Retrospective cohort	11/64	–	M 13.4	M 6.0	M 19.4	–	A (–) GI (–) U (–) W (–) E (–) Anaphylaxis: 0/11	Eg (7) CM (9) So (2) N (6) W (3) Fi (4) O (3)	–	FK (11)	0/11	6/11	– (18–84)
2	Lykavieris et al. (19)	Retrospective cohort	12/121 (10%) IgE-med 12 (100%)	5 (42%)	M 15.9	M 29.1	M 45.0	BA (9) Ala (1) Met (1) O (1)	A (12) GI (9) S&W (4) Anaphylaxis: 5/12	Eg (6) CM (4) P (4) So (4) N (2,1) W (3) Fi (2) Fr (1) L (2) O (1,1,1,1)	10/12	FK (12)	FK>CsA 8/12: resol. 8/8	8/12	M 34.8, (12.0-50.4)
3	Granot et al. (20)	Retrospective cohort	3/30 (10%)	–	–	–	–	BA (17) Met (1) ALF (7) O (5)^*^	A (–) GI (–) U (–) W (–) S (–) EGID (–) Anaphylaxis: 0/3	–	–	FK (3)	–	–	–
4	Levy et al. (9)	Retrospective cohort	4/65 (6%) IgE-med 4 (100%)	2 (50%)	M 31.5	M 36.0	M 67.5	BA (2) Ala (2) Met (1)	A (3) U (3) S (1) Anaphylaxis: 0/4	Eg (2) CM (2) P (2) So (2) N (2) Fi (1) Fr (1) Se (2)	3/4	FK (4)	FK>CsA 2/4: no change 2/2	0/4	– (24.0–48.0)
5	Ozbek et al. (14)	Prospective cohort	6/28 (21%)	2 (33%)	M 10.2	M 9.7	M 19.9	BA (2) Col (2) Met (2)	A (2) GI (5) U (3) EGID (3) Anaphylaxis: 0/6	Eg (6) CM (6) W (5) Fr (1) L (1)	6/6	FK (6)	FK>CsA 2/5: resol. 1/2 FK>Sir 1/3: resol. 1/1	All FA 4/6 Some FA 2/6	M 60 (60.0–60.0)
6	Ozbek and Ozcay (21)	Prospective cohort													
7	Noble et al. (22)	Retrospective cohort	12/78 (15%) IgE-med 10 (83%)	4 (33%)	M 31.8	M 24.3	M 56.1	C (46) Met (11) ALF (8) T (2) O (11)^*^	GI (2) U (8) E (2) EGID (4) Anaphylaxis: 4/12	Eg (5) CM (3) So (1) N (4) W (1) Sh (4)	9/12	FK (12)	–	–	–
8	Brown et al. (23)	Prospective cohort	12/40 (30%) IgE-med 10 (83%)	–	M 9.9	M 15.1	M 25.0	BA (8) ALF (2) T (1) O (1)	A (5) GI (4) U (3) E (1) O (1) EGID (1) Anaphylaxis: 0/12	Eg (7) CM (2) P (4) So (2) N (3) Fi (3) Sh (1) Fr (1) Se (1)	5/12	–	–	–	M 58.6 (–)
9	Shroff et al. (24)	Retrospective cohort	10/176 (6%) IgE-med 10 (100%)	–	–	M 27.1	–	BA (–) C (–) Met (–)	–	Eg (–) CM (–) P (–)	–	FK (10)	–	–	–
10	De Bruyne et al. (10)	Retrospective cohort	13/49 (27%) IgE-med 3 (23%)	–	M 14.5	M 9.3	M 23.8	BA (7) Met (1) ALF (2) C (1) O (2)	A (5) GI (10) U (1) O (3) Anaphylaxis: 1/13	Eg (7) CM (6) P (3) So (3) W (1) Sh (1) Fr (3) O (1)	8/13	–	–	1/13	Md 72 (33.0–188.0)
11	Lee et al. (25)	Prospective cohort	35/93 (38%)	–	–	–	–	BA (67) Ala (4) Met&T (14) ALF (5) O (3)^*^	–	–	–	FK (–)	–	–	–
12	Catal et al. (26)	Retrospective cohort	6/49 (12%) IgE-med 6 (100%)	5 (83%)	M 73.5	M 6.3	M 79.8	C (2) Gen (1) O (3)	A (2) U (3) Anaphylaxis: 2/6	Eg (2) CM (1) P (1) Fr (1) O (1)	0/6	FK (5) CsA (1)	–	–	–
13	Lebel et al. (27)	Retrospective cohort	12/154 (8%) IgE-med 12 (100%)	7 (58%)	M 8.3	M 23.9	M 32.2	BA (10) Met (1) O (1)	A (3) GI (6) U (5) EGID (1) Anaphylaxis: 0/12	Eg (5) CM (5) P (6) N (4) Fi (1) L (2) O (2)	6/12	FK (9) CsA (3)	–	2/12	–
14	Topal et al. (28)	Retrospective cohort	4/29 (14%)	–	–	–	–	C (5) Met (5) ALF (7) O (12)^*^	–	–	–	–	–	–	–
15	Shoda et al. (29)	Retrospective cohort	15/106 (14%)	10 (67%)	M 10.0	M 24.0	M 34.0	BA (11) O (4)	A&U (12) GI (50%) EGID (2) Anaphylaxis: 0/15	Eg (50%)	–	FK (15)	–	–	–
16	Mitsui et al. (30)	Retrospective cohort	42/206 (20%) IgE-med 32 (76%)	20 (48%)	M 9.0^#^ Md 8	M 4.0^#^ Md 3	M 13.0^#^	BA (24) Ala (2) Met (5) ALF (10) O (1)	A&U (26) GI (19)	Eg (25) CM (17) P (2) So (2) W (2) Fi (7) Sh (1) Se (2) O (2)	21/42	FK (42)	–	–	–
17	Marcus et al. (5)	Cross-sectional	17/111 (15%)	–	–	–	–	–	Anaphylaxis: 3/17	–	–	–	–	–	–
18	Mori et al. (31)	Retrospective cohort	7/12 (58%) IgE-med 7 (100%)	–	M 16.1	M 12.9	M 29.0	BA (8) Met (1) Col (3)^*^	A (3) GI (3) U (6) O (2) Anaphylaxis: 3/7	Eg (4) CM (1) P (1) So (1) N (1) W (2) Fi (2) Fr (1) L (1)	5/7	FK (7)	–	3/7	–
19	Sinitkul et al. (32)	Retrospective cohort	25/46 (54%) IgE-med 12 (48%)	17 (68%)	M 16.0	M 13.2^#^ Md 12.2	M 29.2^#^	BA (22) Ala (1) Met (1) ALF (1)	A (8) GI (17) U (5) S (1) E (9) O (2) EGID (4) Anaphylaxis: 3/25	Eg (10) CM (17) P (3) So (14) N (1) W (6) Fi (6) Sh (16) Fr (3) O (1)	22/25	FK (16) FK+ MMF (9)	–	Some FA 5/25	M 72.1^#^ Md 67.4
20	Almaas et al. (7)	Cross-sectional	23/59 (39%) IgE-med 23 (100%)	–	–	M 13.2^#^	–	BA (29) C (11) Met (5) ALF (6) T (5) O (3)^*^	A (18) GI (2) U (4) W&S (10) Severe: 19/23	–	17/23	FK+MMF (–)	–	–	–
21	Bariş et al. (33) *[include pts. from Ozbek et al. (14) and Ozbek and Ozcay (21)]*	Retrospective cohort	19/236 (8%) IgE-med 16 (84%)	7 (37%)	M 7.9	M 14.5	M 22.4	BA (8) C (4) Met (4) ALF (3)	A (9) GI (19) U (12) W (7) EGID (4) Anaphylaxis: 1/19	Eg (14) CM (14) So (2) N (8) W (6) Fi (1) Fr (3) L (8) Se (2) O (2)	17/19	FK (19)	FK>CsA 2/19 FK>Sir 2/19 FK>FK+Sir 2/19	All FA 7/19 Some FA 8/19 None 4/19	M 57.1 (–)
22	Käppi et al. (34)	Cross-sectional	12/43 (28%) IgE-med 11 (92%)	6 (50%)	M 25.0	M 41.0	M 66.0	BA (4) Met (2) ALF (1) T (2) Gen (1) O (2)	A (7) GI (3) U (1) W (1) E (1) O (1) EGID (1) Anaphylaxis: 1/12	Eg (3) CM (3) P (1) So (2) N (3) W (1) Fi (2) Fr (1) L (1)	2/12	FK (11) None (1)	–	4/12	M 123.6 (48.0-201.6)
**Case-control studies**															
23	Maarof et al. (35)	Case-control	7/– IgE-med 7 (100%)	–	M 5.0	M 33.0	M 38.0	BA (7)	A (7) GI (–) U (–) O (2) Anaphylaxis: 0/7	Eg (3) P (3) N (5) Fi (1) L (2) O (1)	7/7	FK (7)	FK>CsA 7/7	7/7	M 79.0 (56.0-117.0)
24	Wisniewski et al. (36)	Case-control	30/352 (9%) IgE-med 19 (63%)	12 (40%)	M 14.0^#^ Md 10.8	M 38.8^#^ Md 12.0	M 52.8^#^	BA (7) C (6) Met (2) ALF (2) T (3)	A&U (12) GI (16) EGID (11) Anaphylaxis: 4/30	Eg (17) CM (18) P (14) So (8) N (7) W (6) Fi (4) Se (4) O (7, 3)	–	FK (27) CsA (3)	–	10/24	M 125.2^#^
25	Nahum et al. (37)	Case-control	8/– IgE-med 8 (100%)	–	M 9.7	–	–	BA (5) ALF (2) O (1)	A (–) U (–) W (–) S (–) Anaphylaxis: ≤ 1	Eg (–) CM (–) P (–) So (–) N (–) Fi (–) Se (–) O (–)	4/8	FK (8)	–	–	M 57.0 (18.0-108.0)
26	Haflidadottir et al. (38)	Case-control	9/– IgE-med 9 (100%)	–	M 8.2^#^ Md 7.2	–	–	BA (11) C (5) Met (1) ALF (3) T (2)^*^	A (–) U (–) W (–) S (–) Anaphylaxis: ≤ 1	Eg (–) CM (–) N (–) Fi (–) Sh (–) Fr (–) O (–)	–	FK (9)	FK+MMF> MMF 1/9: improv. 1/1 FK> MMF 1/9: improv. 1/1	Some 2/9	–
**Case reports and series**															
27	Lacaille et al. (39)	Case report	1/– IgE-med 1 (100%)	1 (100%)	7	5	12	BA (1)	A (1) GI (1) U (1) W (1) Anaphylaxis: 0/1	CM (1)	0/1	FK (1)	0/1	0/1	42.0
28	Inui et al. (40)	Case series	2/– IgE-med 2 (100%)	2 (100%)	M 33.0	M 16.0	M 49.0	Met (2)	A (2) GI (1) Anaphylaxis: 0/2	Fi (2)	0/2	FK (2)	0/2	0/2	–
29	Nowak-Wegrzyn et al. (41)	Case series	6/- IgE-med 6 (100%)	–	M 10.3	M 8.9	M 19.2	BA (6)	A (4) GI (3) U (1) EGID (3) Anaphylaxis: 2/6	Eg (3) CM (2) P (5) So (1) O (1)	3/6	FK (1) FK+MMF (5)	–	–	–
30	Arikan et al. (42)	Case series	2/46 (4%) IgE-med 2 (100%)	–	M 108.0	M 7.5	M 115.5	Met (1) ALF (1)	A&U (2) Anaphylaxis: 0/2	–	–	FK (1) CsA (1)	–	–	–
31	Pacifico et al. (43)	Case report	1/– IgE-med 1 (100%)	1 (100%)	6	2	8	BA (1)	GI (1) U (1) W (1) Anaphylaxis: 0/1	CM (1)	0/1	FK (1)	–	–	–
32	Boyle et al. (44)	Case report	1/– IgE-med 1 (100%)	0 (0%)	19	1	20	BA (1)	U (1) Anaphylaxis: 0/1	Eg (1) P (1)	1/1	FK (1)	–	–	–
33	Yilmaz et al. (45)	Case report	1/– IgE-med 1 (100%)	1 (100%)	8	8	16	BA (1)	A (1) S (1) Anaphylaxis: 0/1	Fr (1)	0/1	FK (1)	FK>CsA>FK 1/1: improv. 1/1	–	–
34	Özdemir et al. (15)	Case report	1/– IgE-med 0 (0%)	1 (100%)	18	7	25	Met (1)	GI (1) EGID (1) Anaphylaxis: 0/1	Eg (1) P (1)	1/1	FK+MMF (1)	–	0/1	24.0
35	Saeed et al. (46)	Case series	3/45 (7%) IgE-med 0 (0%)	0 (0%)	M 44.0	M 20.3	M 64.3	BA (1) Ala (1) Met (1)	GI (3) EGID (3) Anaphylaxis: 0/3	Eg (1) CM (3) W (1) O (1)	1/3	FK (3)	FK>CsA 3/3: no change 3/3	0/3	–
36	Frischmeyer-Guerrerio et al. (47)	Case series	22/– IgE-med 20 (91%)	10 (45%)	M 9.8	M 10.2	M 20.0	BA (13) Met (1) ALF (1) O (7)	A (14) GI (19) U (13) W (9) E (5) O (3) EGID (13) Anaphylaxis: 0/22	Eg (17) CM (19) P (8) So (8) W (8) O (11)	17/22	FK (14) FK+MMF (11)	–	All 2/22 Some 12/22 None 8/22	M 62.8 (8.4–133.2)
37	Cardet et al. (48)	Case report	1/– IgE-med 1 (100%)	1 (100%)	5	9	14	BA (1)	GI (1) U (1) Anaphylaxis: 0/1	Eg (1) P (1) So (1)	1/1	CsA (1)	CsA>FK>FK+ MMF >MMF 1/1: improv. 1/1	–	–
38	Mavroudi et al. (49)	Case series	3/– IgE-med 3 (100%)	2 (67%)	M 7.0	M 74.0	M 81.0	BA (3)	A (1) GI (3) S (1) Anaphylaxis: 0/3	Eg (1) CM (3) Fi (1) Se (1)	1/3	FK (3)	0/3	All 2/3 Some 1/3	M 160.0 (113.0–210.0)
39	Topal et al. (50)	Case report	1/– IgE-med 1 (100%)	1 (100%)	9	0.2	9.2	BA (1)	U (1) S (1) Anaphylaxis: 1/1	CM (1)	0/1	FK (1)	0/1	1/1	20
40	Kehar et al. (51)	Case series	4/– IgE-med 2 (50%)	1 (25%)	M 9.8	M 11.0	M 20.8	BA (2) Col (1) Met (1)	EGID (2) Anaphylaxis: 1/4	Eg (–) CM (–) P (–) So (–) N (–) W (–) Fi (–) Sh (–) L (–) Se (–)	2/4	FK (4)	FK>Sir 4/4: resol. 1/4, improv. 1/4, no change 2/4	1/4	M 44.8 (11.0–60.0)

* *Refers to all pediatric LT patients of the study, including those without FA*.

# *Calculated using Wan et al.'s method (52)*.

*General: FA, food allergy; FU, follow-up; IS, immunosuppression; Improv., improvement; LT, liver transplant; M, mean; Md, median; Mos., months; Pts., patients; Resol., resolution; -, no information provided*.

*Indications to LT: BA, biliary atresia; Ala, Alagille syndrome; C, cholestatic disease; Met, metabolic disease (including alpha-1 antitrypsin deficiency, Wilson disease, hyperoxaluria, Crigler-Najjar); ALF, acute liver failure; T, tumors; Gen, genetic diseases; O, other*.

*Clinical manifestations: U, urticaria or rash; A, angioedema; W, wheezing; S, stridor; GI, gastrointestinal symptoms (vomit, diarrhea, abdominal pain); E, eczema (triggered by ingestion of culprit food); O, other; EGID, eosinophilic gastrointestinal disease*.

*Culprit foods: Eg, egg; CM, cow's milk; P, peanut; Fi, fish; So, soy; N, nuts; W, wheat; Fr, fruits; L, legumes; Se, sesame; Sh, shellfish; O, other*.

*Immunosuppression: FK, Tacrolimus; CsA, Cyclosporine A; MMF, Mycophenolate; Sir, Sirolimus; O, other*.

### Statistical Analysis

Finally, findings from included individual studies were summarized and grouped together to perform a quantitative synthesis of results as to address the above-mentioned research questions. Due to the expected heterogeneity between studies, it was *a priori* determined that a meta-analysis could not be performed.

Descriptive statistics were conducted using Microsoft Excel (Redmond, WA) and employed to summarize the characteristics of the data set. Categorical variables were described as numbers with percentage, whereas continuous variables were calculated as weighted mean and standard deviation. Missing data points were addressed by adjusting the denominator for the number of non-missing items.

When the mean was unavailable, the method of Wan et al. was used to estimate it using the median, the first and third quartiles and the sample size (52). Odds ratios (ORs) were calculated with 95% confidence intervals (CIs).

## Results

### Characteristics of Included Studies

As shown in the PRISMA flow diagram, 40 eligible articles were included in the systematic revision (Figure 1). Table 1 summarizes the main characteristics of the included studies. Only 4 studies were prospective, while the remaining were either retrospective (*n* = 33) or cross-sectional (*n* = 3).

The number of pediatric patients with *de novo* FA in each paper varied between 1 and 42, for a cumulative population of 397 LT recipients with newly-onset FA. 80.8% (252/312) of subjects had IgE-mediated FA.

Data obtained from the systematic review are reported in the next paragraphs, each of which is dedicated to answering one of the six research questions related to *de novo* FA after pediatric LT.

### What Is the Epidemiology of *de novo* Post-liver Transplant Food Allergy?

When taken individually, the studies included in our review report an extremely variable prevalence of new-onset FA, ranging from 4 to 58%, probably due to the heterogeneity and to the relatively small samples of enrolled patients (Table 1). By combining all the studies that included a control population of non-allergic LT children, the prevalence of FA among pediatric LT recipients amounts to 14.9% (329 subjects with *de novo* FA/2210 LT recipients).

As regards FA onset, the average time interval between LT and the first clinical allergic manifestation resulted equal to 18.1 months (±12.8, range 0.2–74.0) with a mean age at allergy onset of 33.8 months (±19.2, range 8.0–115.5).

### What Are the Risk Factors?

#### Recipient's Age at the Time of Transplant

Many studies investigated whether the recipient's age at the time of LT was a risk factor for the onset of *de novo* FA. The single prospective study addressing this issue in a group of 28 pediatric patients showed that FA mainly occurs in children younger than 1 year of age (14). As for the retrospective studies, Topal et al. (28) showed that children were more likely to develop FA after LT than adults, although there was no difference in the onset of other atopic disorders, such as asthma or allergic rhinitis. Among children, the likelihood of developing new-onset FA resulted inversely proportional to the age at transplantation, with the greatest risk under the age of two (5, 8–10, 24, 25, 27, 32, 33, 36).

Considering the population of transplanted children included in this systematic review as a whole, the mean age at transplant of patients who developed *de novo* FA was 15.1 months (±13.2, range 5.0–108.0), with 82.5% (188/228) of these children undergoing LT before the age of 2 years old.

#### Recipient's Personal and Family History of Atopy

Several studies evaluated the association between a personal or family history of atopic disorders before LT and the occurrence of FA after LT. No evidence of a significant correlation was observed by the prospective study of Ozbek et al. (14). However, the opposite was reported by other retrospective and cross-sectional studies, that recognized both in the familiar and in the personal history to atopy a significant risk factor for the development of *de novo* FA (5, 32). Moreover, the presence of eczema at the time of LT was strongly related to new onset of IgE-mediated FA (30).

In our analysis, one-third (36/113, 31.9%) of children with *de novo* FA had atopic diseases prior to LT, defined as eczema, asthma, or allergic rhinitis. Similarly, a familiar history of atopy was reported in 34.3% (37/108) of subjects in which this information was available.

#### Donor's Characteristics

Few studies have investigated whether the donor's allergic status plays a role in the onset of *de novo* FA after LT, as this information is often unavailable to physicians and researchers. The only prospective study addressing this issue found that none of the donors had a history of FA or positive food specific sIgE or positive skin prick test prior to transplantation (14). Similarly, the retrospective study by Sinitkul et al. (32) did not observe any difference in terms of donor atopic status between allergic and non-allergic LT recipients. However, the results of both these studies are hampered by the low number of participants (14, 32).

In our analysis, 16 out of 64 children with FA (25.0%), for whom information on the donor's allergic status was available, received the liver graft from a subject with a history of allergic disease.

A single retrospective study reported a significantly lower donor age in the FA group than in the non-FA group, but patients' data are not available for further analysis (10). On the contrary, in a multivariate analysis performed by Mitsui et al. (30) and in the case-control study by Wisniewski et al. (36) the donor's age was not associated with a higher risk of development of FA after LT.

#### Immunosuppression

Many authors have investigated the causal link between the onset of *de novo* FA and immunosuppression. Tacrolimus has been strongly implicated in the development of FA by a large number of studies (9, 15, 19, 25, 27, 41, 42, 46, 54). Consistently, 96.6% (283/293) of the pediatric LT recipients included in our analysis, for whom details on immunosuppression were provided, developed FA while on treatment with tacrolimus.

Despite being a calcineurin inhibitor as tacrolimus, cyclosporine has not been consistently associated with an increased risk of FA compared to the general population (14, 27, 42). Indeed, the prevalence of *de novo* FA in the population of children treated with cyclosporine, obtained from the combination of available studies in which immunosuppressive therapy of pediatric LT patients was specified, resulted 3.4% (4/119), much lower than that of subjects receiving tacrolimus, equal to 20.1% (137/683). Thereby, the odds of developing FA were higher among children treated with tacrolimus, compared with those receiving cyclosporine (OR 7.21, 95% CI 2.62–19.89, *P* = 0.0001). The role of tacrolimus in the pathogenesis of FA is further supported by the resolution or improvement of post-transplant allergies described after switch from tacrolimus to cyclosporine or sirolimus (19, 21, 35, 51, 54).

#### Epstein-Barr Virus Infection

The only prospective study on this subject reported that two-thirds of food-allergic pediatric LT recipients developed Epstein-Barr virus (EBV) infection before the onset of FA, but this result is hampered by the very small population of the study, consisting of only 6 subjects (14). A cross-sectional study identified post-transplant EBV infection as an independent risk factor for the development of allergy or autoimmunity in a multivariate analysis (5). Several retrospective studies also suggested the role of EBV in the development of post-transplant FA, ascribing it to the virus-induced immunological imbalance toward Th2 responses, but also acknowledging that a causal role for immunosuppression could not be excluded, as both FA and EBV infection may be favored by tacrolimus (14, 25, 32, 33). As regards our population, EBV was positive in 38.0% (35/92) of food allergic LT recipients whose virus status was available for analysis.

#### Other Risk Factors

A prospective study found that the total eosinophil count was higher in children with FA compared to non-allergic ones (14). However, eosinophilia has been considered more as a marker than as a cause of FA and has itself been associated with the use of calcineurin inhibitors (14, 27, 33).

Other clinical elements have been evaluated as possible risk factors for the development of post-LT FA, but their role has not been clearly proven. They include post-transplant lymphoproliferative disease (25), graft rejection (34), autoantibodies levels (34), biliary atresia as indication for LT (29), and female genre (5, 55). As for the latter, we did not find any significant difference between sexes in the studies reporting this information. Indeed, males and females were equally represented among the whole population of allergic children (116 females/230 patients, 50.4%).

Factors not found to be associated with a higher risk for *de novo* FA development included steroidal treatment, acute rejection(s), organ type (living donor *vs*. cadaveric), donor/recipient blood type and compatibility (5, 36).

### What Are the Most Implicated Foods?

34/40 studies investigated the foods implicated in *de novo* FA. Overall, the most frequent culprit foods were egg (at least 142/267 children in which trigger foods were specified, 53.2%), cow's milk (132/267, 49.4%), peanuts (60/267, 22.5%), soy (53/267, 19.9%), nuts (47/267, 17.6%), wheat (40/267, 15.0%), fish (37/267, 13.9%), shellfish (23/267, 8.6%), fruits (16/267, 6.0%), legumes (16/267, 6.0%), and sesame (12/267, 4.5%).

One hundred and fifty three subjects out of 249 (61.5%) were reported as affected by multiple concomitant food allergies.

### What Are the Clinical Manifestations?

A subgroup analysis of the included studies was performed as to evaluate the diverse clinical manifestations of *de novo* FA, which were reported in 37/40 of included papers.

At least 15.9% (27/170) of children with IgE-mediated FA presented with anaphylaxis. None of the studies reported fatal events.

Cutaneous manifestations were the most common. Particularly, angioedema was reported in more than half of patients (52.3%, 157/300) for which clinical information was available, and urticaria in 122/293 (41.6%). Gastrointestinal manifestations (diarrhea, vomit, abdominal pain) were described in 143/293 (48.8%) subjects, while respiratory symptoms, wheezing and stridor were observed in 11.0% (33/300) and 6.3% (19/300), respectively. EGID was reported in 16.7% (50/300) of food allergic transplanted children.

### What Is the Prognosis of *de novo* Post-liver Transplant Food Allergy?

Several authors evaluated the long-term prognosis of *de novo* FA overtime. Length of follow-up varied greatly among studies, ranging from a minimum of 6.5 months to a maximum of 17 years, with a mean of 71.0 ± 35.4 months. Only a minority of patients were eventually able to follow an unrestricted diet, while a larger part of patients was able to improve symptoms or outgrow allergy to at least some of the involved foods at a variable time after transplant (32, 33, 36). Younger age at LT, high-risk EBV status (i.e., virally naïve recipient plus EBV positive donor), positive family history of atopy, and eosinophilia have been associated to more persistent FA (25, 49). The normalization or improvement of specific IgEs and skin prick tests was used to guide the timing of oral food challenges as well as the reintroduction of culprit foods into the diet (14, 33, 35, 49).

In our analysis, 27.0% (54/200) of patients with FA outgrew all their food allergies, while an additional 14.0% (28/200) of children were able to reintroduce at least some of the culprit foods.

### What Are the Possible Therapeutic Strategies?

Our systematic review of the literature did not retrieve any high-evidence studies, such as prospective or randomized-controlled trials, related to the treatment of *de novo* FA. In several retrospective case reports and small case series a shift in immunosuppressive therapy was attempted as a strategy to promote the reacquisition of oral tolerance to food allergens. A switch from tacrolimus to cyclosporine was reported by 8 studies and led to successful reintroduction of food allergens in 71.4% of patients (15/21, excluding duplicates). Similarly, the switch from tacrolimus to mycophenolate or the addition of mycophenolate to tacrolimus led to the reduction of the biomarkers of Th2 activation (including total and specific IgEs), along with the improvement or the resolution of FA in 3 out of 3 children (38, 48). The introduction of the mTOR inhibitor sirolimus was reported as effective in the treatment of post-transplant immune mediated disorders, such as autoimmune cytopenia, but had mixed results as regards the improvement of FA, reported in 3 out of 5 patients (14, 33, 51). Finally, reduction of tacrolimus dosage, when reported, did not result in the reintroduction of the allergenic foods (19, 40).

## Discussion

We performed a systematic review of the literature in order to provide clinicians with evidence-based information regarding six topics relevant to the management of *de novo* FA after pediatric LT, namely epidemiology, risk factors, culprit food allergens, clinical manifestations, prognosis, and treatment.

**Figure 2:**
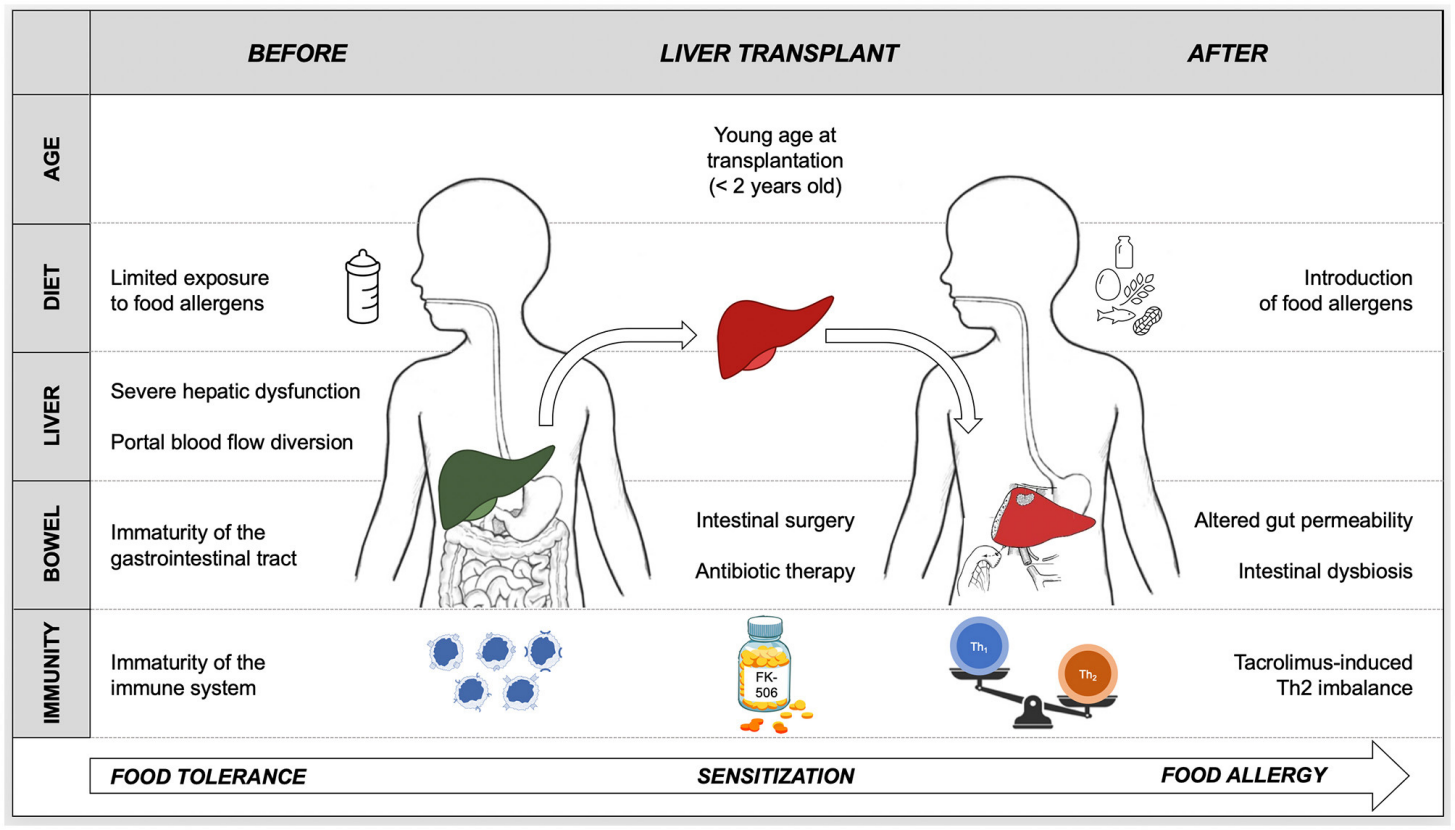
Proposed pathogenesis of *de novo* food allergy in pediatric liver transplant recipients based on current available knowledge. Oral tolerance is the physiologic response to food allergens that takes place during infancy and continues throughout life. A breakdown in this process results in the sensitization to food antigens and in the progression to food allergy. Several factors concur in determining the loss of tolerance to food allergens and in triggering the onset of allergy after pediatric liver transplantation. Tacrolimus-based immunosuppression plays a role in food sensitization by inducing an immunological imbalance toward Th2 responses. The likelihood of developing food allergy is inversely proportional to the age at liver transplantation, hence young age must facilitate the breakdown of oral tolerance. The immaturity of the immune system and of the gastrointestinal tract, combined with the liver dysfunction, the diversion of portal blood flow into the systemic circulation and with the delayed introduction of solid allergenic foods before transplant, hamper oral tolerance acquisition. The disruption of the intestinal barrier with consequent increased gut permeability, induced by surgery, antibiotic-induced dysbiosis and drugs (e.g., tacrolimus), causes a massive exposure of food allergens to an “imbalanced” immune system.

As concerns epidemiology, the prevalence of *de novo* FA in pediatric liver transplant recipients obtained from our analysis of the literature was 15%. This result is not only significantly higher in comparison to the prevalence of FA documented in the general pediatric population (about 1.5–3.5% of objectively confirmed FA) (56), but also far superior than that reported in the recipients of other kinds of solid organs (e.g., about 5% and <1% in heart and kidney transplant recipients, respectively) (5, 10). Little is known about the pathogenetic mechanisms leading to FA in transplant recipients. Several factors may explain the increased susceptibility toward FA observed in pediatric LT patients (Figure 2). Firstly, the liver has a central role in the acquisition of immune tolerance to dietary allergens, as demonstrated by the fact that a severe hepatic dysfunction and the diversion of portal blood into the systemic circulation have been associated with an increased sensitization to food allergens (23). Secondly, intestinal surgery, such as creation of a Roux-en-Y loop at the time of LT, in association with the use of postoperative antibiotics, may alter intestinal permeability and modify the gut microbiota, thereby increasing the risk of post-operative FA (10).

Our data also confirm that the likelihood of developing FA is inversely proportional to the age at LT, with the highest risk in the first 2 years of life. This is consistent with the finding that, after LT, atopic disorders in general and FA in particular develop more frequently in children than in adults (28). Several authors have speculated that the immaturity of the immune system and of the gastrointestinal tract may be responsible for the increased likelihood of sensitization to food allergens in younger subjects (6). In addition, the restrictive diet (predominantly based on artificial formula or parenteral nutrition) that often precedes LT in infants with end stage liver disease may favor the onset of FA by limiting the exposure to dietary allergens at a critical age window for achieving immune tolerance toward foods (6, 31, 36).

Other than age at LT, other elements have been investigated as potential risk factors for the onset of *de novo* FA. A personal or familiar history of allergic disorders of the recipient and a positive allergic status of the donor were present in only one-third and one-fourth of subjects, respectively. This, coupled with the fact that the passive transfer of FA from the donor to the recipient has been seldom documented in children, supports that, in pediatric LT recipients, the emergence of food sensitization occurs most often regardless of donor and recipient allergic status (23).

The pathogenetic role of calcineurin inhibitors in the onset of post-transplant FA has been broadly investigated. Due to the extensive use of tacrolimus compared to cyclosporine and the small number of patients enrolled in individual studies, few authors were able to discriminate whether the onset of FA was associated with the use of the pharmacologic class of calcineurin inhibitors or with tacrolimus *per se*, favoring the latter hypothesis. The data obtained from our systematic review demonstrate that tacrolimus is significantly more associated than cyclosporine with the onset of *de novo* FA after pediatric LT. Although calcineurin inhibitors share the same mechanism of action, tacrolimus is 10–100 times more active than cyclosporine in suppressing T- and B-cell responses and in reducing IL-2 production, and this may favor a stronger immunological imbalance toward Th2 responses (27, 54). Furthermore, tacrolimus, but not cyclosporine, increases intestinal permeability and is thus responsible for an amplified immunological exposure to food allergens (16).

Culprit foods of *de novo* FA do not differ significantly from those usually implicated in otherwise healthy children with FA (1). Similarly, a significant proportion of patients had allergies to multiple foods (57).

More than 15% of children with *de novo* IgE-mediated FA suffered from anaphylaxis. The prevalence of anaphylaxis in our whole study population was 1.4% (23/1,647 patients considering available data), slightly higher than that of food-induced anaphylaxis in the general pediatric population, ranging from 0.3 to 1.2% (58).

No study has identified markers able to predict the onset of *de novo* food allergy in LT recipients. Skin prick tests and serum food-specific IgE (sIgE) indicate the sensitization toward food allergens, but do not necessarily correspond to the presence of clinical FA (1). Moreover, no strategies able to prevent the development of FA after LT have been identified to date and the avoidance of exposure could further increase the risk of sensitization to food allergens (1).

Once arisen, FA tend to persist overtime, with only a minority of patients being able to resume an unrestricted diet. To date, the only effective treatment of *de novo* FA consists, as for allergies arising in non-transplanted subjects, in the avoidance of allergen ingestion and in the prompt pharmacologic treatment in case of allergic reaction (1). As demonstrated by case reports and small case series, the discontinuation of tacrolimus in favor of other immunosuppressive drugs, such as cyclosporine and mycophenolate, or the therapeutic association of tacrolimus with mycophenolate might reduce the immunological imbalance toward Th2 responses, thus leading to the resolution or the improvement of FA. However, before this strategy can be routinely recommended, prospective, controlled studies are needed to evaluate the efficacy and the safety of this therapeutic approach, which, at present, should be reserved for patients with life-threatening allergic reactions or with multiple FA severely limiting their quality of life.

The main limitation of our study relies in the low level of evidence of the vast majority of available articles. Indeed, the retrospective nature of most of the studies, together with their small number of patients, account for the marked heterogeneity of the results and limit the possibility of drawing definitive conclusions. Particularly, very few studies have specifically investigated the characteristics of the donors, possibly hampering our ability to identify donor or graft-related risk factors. However, our work has several strengths. To the best of our knowledge, this is the only systematic review in literature on the subject of *de novo* FA after pediatric LT. Moreover, by combining data from all relevant studies, we obtained the largest available population of transplanted food-allergic children, so that we could answer to clinically relevant questions with the best possible evidence.

In conclusion, *de novo* FA develops in 15% of children after LT, especially in the first 2 years after transplant, with higher risk related to younger age at transplant (especially under 2 years of age) and tacrolimus immunosuppression. FAs cause anaphylaxis in 15% of subjects with IgE-mediated reactions and are often multiple. The majority of patients do not spontaneously outgrow their symptoms, and, to date, no treatment has been clearly proven to resolve the immunological imbalance responsible for the onset of *de novo* FA after LT. Prospective multicenter studies are needed to confirm these findings, guide the risk-based stratification of pediatric LT recipients, and provide therapeutic strategies for children with *de novo* FA.

## Data Availability Statement

The original contributions presented in the study are included in the article/Supplementary Material, further inquiries can be directed to the corresponding author/s.

## Author Contributions

CB conceptualized and designed the study, provided support with the search strategy, screened databases for eligible studies, and reviewed the manuscript. EA, SG, MCu, and FC undertook data collection and analysis. LB and MCa drafted the manuscript. GF and PG critically reviewed the manuscript. All authors read and approved the final manuscript as submitted.

## Conflict of Interest

The authors declare that the research was conducted in the absence of any commercial or financial relationships that could be construed as a potential conflict of interest.

## Publisher's Note

All claims expressed in this article are solely those of the authors and do not necessarily represent those of their affiliated organizations, or those of the publisher, the editors and the reviewers. Any product that may be evaluated in this article, or claim that may be made by its manufacturer, is not guaranteed or endorsed by the publisher.

## Abbreviations

CsA: cyclosporine A
EBV: Epstein-Barr virus
EGID: eosinophilic gastrointestinal disorders
FA: food allergy
LT: liver transplant
MMF: mycophenolate.
