# Supplementary material for: De novo Food Allergy After Pediatric Liver Transplantation: A Systematic Review
Source: Front Pediatr. 2022 May 12;10:885942. doi: 10.3389/fped.2022.885942 (PMC9134120; doi:10.3389/fped.2022.885942)
Supplement: Supplementary file 1 [file Data_Sheet_1.PDF]

Supplementary Material

Supplementary Table S1. Search strategy employed in PubMed.

No limits activated	
#1	MeSH descriptor Food Hypersensitivity (Result #22,477)
#2	MeSH descriptor Liver Transplantation (Result #61,198)
#3	Title/Abstract Food Allergy (Result #10,640)
#4	#3 with limits added for Child: birth-18 years (Result #4,675)
#5	Title/Abstract Liver Transplantation (Result #56,034)
#6	#5 with limits added for Child: birth-18 years (Result #11,493)
#7	(#1) AND (#2) (Result #60)
#8	(#4) AND (#6) (Result #33)
"Food Hypersensitivity"[MeSH Terms] AND "Liver Transplantation"[MeSH Terms]<br>("food allergy"[Title/Abstract] AND "liver transplantation"[Title/Abstract]) AND (allchild)[Filter]	

Supplementary Table S2. Studies excluded from the systematic revision and rationale for exclusion.

	**Study**	**Reason for exclusion**
1	Romero et al., 2003	No FA cases
2	Ozbek et al., 2015	Letter to the editor, no further data compared to 2009's paper
3	Guidi et al., 2007	Insufficient data
4	Eiwegger et al., 2010	Lack of patients' data
5	Blanchard et al., 2003	Unmet diagnostic requirements for FA
6	Narumoto et al., 2017	Unproven FA
7	Parashette et al., 2013	Unmet diagnostic requirements for FA

Supplementary Table S3. Application of the National Institutes of Health (NIH) Study Quality Assessment Tools to the included studies.

**NIH Quality Assessment Tool for Observational Cohort and Cross-Sectional Studies**	Q1 Was the research question or objective in this paper clearly stated?	Q2 Was the study population clearly specified and defined?	Q3 Was the participation rate of eligible persons at least 50%?	Q4 Were all the subjects selected or recruited from the same or similar populations (including the same time period)? Were inclusion and exclusion criteria for being in the study prespecified and applied uniformly to all participants?	Q5 Was a sample size justification, power description, or variance and effect estimates provided?	Q6 For the analyses in this paper, were the exposure(s) of interest measured prior to the outcome(s) being measured?	Q7 Was the timeframe sufficient so that one could reasonably expect to see an association between exposure and outcome if it existed?	Q8 For exposures that can vary in amount or level, did the study examine different levels of the exposure as related to the outcome (e.g., categories of exposure, or exposure measured as continuous variable)?	Q9 Were the exposure measures (independent variables) clearly defined, valid, reliable, and implemented consistently across all study participants?	Q10 Was the exposure(s) assessed more than once over time?	Q11 Were the outcome measures (dependent variables) clearly defined, valid, reliable, and implemented consistently across all study participants?	Q12 Were the outcome assessors blinded to the exposure status of participants?	Q13 Was loss to follow-up after baseline 20% or less?	Q14 Were key potential confounding variables measured and adjusted statistically for their impact on the relationship between exposure(s) and outcome(s)?	**Total quality score**
Prabhakaran et al., 1999	Y	Y	Y	Y	N	Y	Y	N	Y	NA	N	N	NA	N	Fair
Lykavieris et al., 2003	Y	Y	Y	Y	N	Y	Y	Y	Y	NA	Y	N	NA	N	Good
Granot et al., 2006	Y	Y	Y	Y	N	Y	Y	N	Y	NA	Y	N	NA	N	Fair
Levy et al., 2009	Y	Y	Y	Y	N	Y	Y	N	Y	NA	Y	N	NA	N	Fair
Ozbek et al., 2009	Y	Y	Y	Y	N	Y	Y	NR	Y	NA	Y	N	Y	N	Good
Ozbek et al., 2015	Y	Y	Y	Y	N	Y	Y	N	Y	NA	Y	N	Y	N	Good
Noble et al., 2011	Y	Y	Y	Y	N	Y	Y	N	Y	NA	Y	N	N	N	Fair
Brown et al., 2012	Y	Y	Y	Y	N	Y	Y	NR	Y	NA	Y	N	Y	NR	Good
Shroff et al., 2012	Y	Y	Y	Y	N	Y	NR	N	Y	NA	Y	N	NA	N	Fair
De Bruyne et al., 2013	Y	Y	Y	Y	N	Y	Y	N	Y	NA	Y	N	Y	N	Good
Lee et al., 2013	Y	Y	Y	Y	N	Y	Y	Y	Y	NA	N	N	Y	N	Good
Catal et al., 2014	Y	Y	Y	Y	N	Y	Y	Y	Y	NA	Y	N	NA	N	Good
Lebel et al., 2014	Y	Y	Y	Y	N	Y	Y	N	Y	NA	Y	N	Y	N	Good
Topal et al., 2014	Y	Y	Y	Y	N	Y	NR	N	Y	NA	N	N	NA	N	Fair
Shoda et al., 2015	Y	Y	NA	Y	N	Y	NR	NA	Y	NA	Y	N	NA	N	Fair
Mitsui et al., 2017	Y	Y	Y	Y	N	Y	Y	Y	Y	NA	Y	N	Y	Y	Good
Marcus et al., 2018	Y	Y	Y	Y	N	N	N	N	Y	NA	N	N	Y	Y	Fair
Mori et al., 2018	Y	Y	Y	Y	N	Y	Y	N	Y	NA	Y	N	Y	N	Good
Sinitkul et al., 2018	Y	Y	Y	Y	N	Y	Y	Y	Y	NA	Y	N	Y	Y	Good
Almaas et al., 2019	Y	Y	Y	Y	N	N	N	Y	Y	Y	N	N	Y	Y	Good
Barış et al., 2019	Y	Y	Y	Y	N	Y	Y	Y	Y	NA	Y	N	Y	N	Good
Käppi et al., 2019	Y	Y	Y	Y	N	N	N	N	Y	NA	Y	N	Y	N	Fair

**NIH Quality Assessment Tool for Case-Control Studies**	Q1 Was the research question or objective in this paper clearly stated and appropriate?	Q2 Was the study population clearly specified and defined?	Q3 Did the authors include a sample size justification?	Q4 Were controls selected or recruited from the same or similar population that gave rise to the cases (including the same timeframe)?	Q5 Were the definitions, inclusion and exclusion criteria, algorithms or processes used to identify or select cases and controls valid, reliable, and implemented consistently across all study participants?	Q6 Were the cases clearly defined and differentiated from controls?	Q7 If less than 100 percent of eligible cases and/or controls were selected for the study, were the cases and/or controls randomly selected from those eligible?	Q8 Was there use of concurrent controls?	Q9 Were the investigators able to confirm that the exposure/risk occurred prior to the development of the condition or event that defined a participant as a case?	Q10 Were the measures of exposure/risk clearly defined, valid, reliable, and implemented consistently (including the same time period) across all study participants?	Q11 Were the assessors of exposure/risk blinded to the case or control status of participants?	Q12 Were key potential confounding variables measured and adjusted statistically in the analyses? If matching was used, did the investigators account for matching during study analysis?	**Total quality score**
Maarof et al., 2011	Y	CD	N	Y	Y	Y	CD	NR	Y	Y	N	N	Fair
Wisniewski et al., 2012	Y	CD	N	Y	Y	Y	CD	N	Y	Y	N	N	Fair
Nahum et al., 2015	Y	CD	N	Y	N	Y	CD	N	Y	Y	N	N	Fair

Haflidadottir et al., 2020	Y	CD	N	Y	N	Y	CD	N	Y	Y	N	N	Fair

**NIH Quality Assessment Tool for Case Series Studies**	Q1 Was the study question or objective clearly stated?	Q2 Was the study population clearly and fully described, including a case definition?	Q3 Were the cases consecutive?	Q4 Were the subjects comparable?	Q5 Was the intervention clearly described?	Q6 Were the outcome measures clearly defined, valid, reliable, and implemented consistently across all study participants?	Q7 Was the length of follow-up adequate?	Q8 Were the statistical methods well-described?	Q9 Were the results well-described?	**Total quality score**
Lacaille et al., 1997	N	N	NA	NA	NA	NA	Y	NA	Y	Poor
Inui et al., 1999	N	N	NR	Y	NA	Y	NR	NA	Y	Poor
Nowak-Wegrzyn et al., 2001	N	Y	NR	Y	NA	Y	NR	NA	Y	Fair
Arikan et al., 2003	N	Y	Y	Y	NA	Y	NR	NA	Y	Fair
Pacifico et al., 2003	N	N	NA	NA	NA	Y	NR	NA	Y	Poor
Boyle et al., 2005	N	Y	Y	N	NA	Y	NR	NA	Y	Fair
Yilmaz et al., 2005	N	N	NA	NA	Y	Y	Y	NA	Y	Fair
Özdemir et al., 2006	N	N	N	N	NA	Y	Y	NA	Y	Poor
Saeed et al., 2006	N	Y	NA	Y	Y	Y	NR	NA	Y	Good
Frischmeyer-Guerrerio et al., 2008	Y	Y	NA	Y	Y	Y	NA	N	Y	Good
Cardet et al., 2012	Y	N	NA	NA	Y	Y	NA	NA	Y	Fair
Mavroudi et al., 2012	Y	Y	NR	Y	NA	Y	Y	NA	Y	Good
Topal et al., 2013	Y	N	NA	NA	NA	Y	Y	NA	Y	Fair
Kehar et al., 2020	Y	Y	Y	Y	Y	Y	Y	NA	Y	Good

Quality was rated as poor (0–4 for cohort and case-control studies, 0-3 for case-series), fair (5–8 out of 14 questions for cohort studies, 5-7 for case-control studies, 4-5 for case-series), or good (9–14 for cohort studies, 8-12 for case-control studies, 6-9 for case series). CD: cannot determine; NA: not applicable, NR: not reported.

Supplementary Table S4. Studies from which relevant data were extracted to answer to each research question.

		Epidemiology	Risk factors: Age at transplant	Risk factors: personal history of atopy before LT	Risk factors: familiar history of atopy	Risk factors: donor's characteristics	Risk factors: immunosuppression	Risk factors: EBV	Implicated foods	Clinical manifestations	Prognosis	Therapeutic strategies
	**Cohort and cross-sectional studies**											
1	Prabhakaran et al., 1999	+	+	-	-	-	+	-	+	+	+	-
2	Lykavieris et al., 2003	+	+	+	+	-	+	-	+	+	+	+
3	Granot et al., 2006	+	-	-	-	-	+	-	-	+	-	-
4	Levy et al., 2009	+	+	-	-	-	+	-	+	+	+	+
5	Ozbek et al., 2009	+	+	+	+	+	+	+	+	+	+	+
6	Ozbek et al., 2015											
7	Noble et al., 2011	+	+	-	-	-	+	-	+	+	-	-
8	Brown et al., 2012	+	+	-	-	-	-	-	+	+	-	-
9	Shroff et al., 2012	+	-	-	-	-	+	-	+	-	-	-
10	De Bruyne et al., 2013	+	+	-	-	+	+	-	+	+	+	-
11	Lee et al., 2013	+	-	-	-	-	-	-	-	-	-	-
12	Catal et al., 2014	+	+	-	-	+	+	+	+	+	-	-
13	Lebel et al., 2014	+	+	-	-	-	+	-	+	+	+	-
14	Topal et al., 2014	+	-	-	-	-	-	-	-	-	-	-
15	Shoda et al., 2015	+	+	-	-	-	+	-	+	+	-	-
16	Mitsui et al., 2017	+	-	+	+	+	-	+	+	+	-	-
17	Marcus et al., 2018	+	-	-	-	-	-	-	-	+	-	-
18	Mori et al., 2018	+	+	+	-	-	+	-	+	+	+	-
19	Sinitkul et al., 2018	+	+	+	+	+	+	+	+	+	+	-
20	Almaas et al., 2019	+	-	-	-	-	-	-	-	+	-	-
21	Barış et al., 2019	+	+	-	-	+	+	+	+	+	+	-
22	Käppi et al., 2019	+	+	-	-	-	+	-	+	+	+	-
	**Case-control studies**											
23	Maarof et al., 2011	+	+	-	+	-	+	-	+	+	+	+
24	Wisniewski et al., 2012	+	+	-	-	+	+	+	+	+	+	-
25	Nahum et al., 2015	+	+	-	-	-	+	-	+	+	-	-
26	Haflidadottir et al., 2020	+	+	-	-	-	+	-	+	+	-	+
	**Case series and case reports**											
27	Lacaille et al., 1997	+	+	+	+	+	+	-	+	+	+	-
28	Inui et al., 1999	+	+	+	+	+	+	-	+	+	+	-
29	Nowak-Wegrzyn et al., 2001	+	+	-	+	+	+	-	+	+	-	-
30	Arikan et al., 2003	+	+	+	+	-	+	-	-	+	-	-
31	Pacifico et al., 2003	+	+	-	+	-	+	-	+	+	-	-
32	Boyle et al., 2005	+	+	+	-	+	+	-	+	+	-	-
33	Yilmaz et al., 2005	+	+	-	-	-	+	+	+	+	-	+
34	Özdemir et al., 2006	+	+	+	+	-	+	-	+	+	+	-
35	Saeed et al., 2006	+	+	-	-	-	+	-	+	+	+	+
36	Frischmeyer-Guerrerio et al., 2008	+	+	-	-	-	+	-	+	+	+	-
37	Cardet et al., 2012	+	+	-	+	-	+	-	+	+	-	+
38	Mavroudi et al., 2012	+	+	+	+	+	+	-	+	+	+	-
39	Topal et al., 2013	+	+	+	+	+	+	+	+	+	+	-
40	Kehar et al., 2020	+	+	+	+	-	+	-	+	+	+	+
